# Endocannabinoid-Dependent Modulation of Phasic Dopamine Signaling Encodes External and Internal Reward-Predictive Cues

**DOI:** 10.3389/fpsyt.2014.00118

**Published:** 2014-09-01

**Authors:** Jennifer M. Wenzel, Joseph F. Cheer

**Affiliations:** ^1^Department of Anatomy and Neurobiology, University of Maryland School of Medicine, Baltimore, MD, USA; ^2^Department of Psychiatry, University of Maryland School of Medicine, Baltimore, MD, USA

**Keywords:** dopamine, cannabinoids, reward-seeking behavior, cues, endocannabinoids, fixed interval

## Abstract

The mesolimbic dopamine (DA) system plays an integral role in incentive motivation and reward seeking and a growing body of evidence identifies signal transduction at cannabinoid receptors as a critical modulator of this system. Indeed, administration of exogenous cannabinoids results in burst firing of DA neurons of the ventral tegmental area and increases extracellular DA in the nucleus accumbens (NAcc). Implementation of fast-scan cyclic voltammetry (FSCV) confirms the ability of cannabinoids to augment DA within the NAcc on a subsecond timescale. The use of FSCV along with newly developed highly selective pharmacological compounds advances our understanding of how cannabinoids influence DA transmission and highlights a role for endocannabinoid-modulated subsecond DAergic activation in the incentive motivational properties of not only external, but also internal reward-predictive cues. For example, our laboratory has recently demonstrated that in mice responding under a fixed-interval (FI) schedule for food reinforcement, fluctuations in NAcc DA signal the principal cue predictive of reinforcer availability – time. That is, as the interval progresses, NAcc DA levels decline leading to accelerated food seeking and the resulting characteristic FI scallop pattern of responding. Importantly, administration of WIN 55,212-2, a synthetic cannabinoid agonist, or JZL184, an indirect cannabinoid agonist, increases DA levels during the interval and disrupts this pattern of responding. Along with a wealth of other reports, these results illustrate the role of cannabinoid receptor activation in the regulation of DA transmission and the control of temporally guided reward seeking. The current review will explore the striatal beat frequency model of interval timing as it pertains to cannabinoid signaling and propose a neurocircuitry through which this system modulates interoceptive time cues.

## Introduction

A wealth of psychology research has documented an integral role for environmental stimuli in guiding reward seeking behavior. Stimuli that repeatedly occur in conjunction with the presentation of reinforcers (for the sake of simplicity, “rewards”), themselves gain incentive value as reward predictors and work to motivate behavior (1). For example, the neutral auditory stimulus of a can of cat food being opened, through its repeated pairing with food delivery, serves as an incentive stimulus to the cat that is predictive of food-related stimuli (e.g., taste) and energizes the cat’s approach toward the food bowl. Of course, external motivational cues (e.g., sound of the can opener) frequently interact with internal cues (e.g., hunger) to guide behavior – i.e., if the cat is not hungry the likelihood that it will approach the food bowl is greatly reduced (2, 3). Further, through repeated pairing external cues may gain the ability to elicit internal cues, such as hunger (4) or the initiation of an internal clock that works to predict reward availability (5). While conditioned environmental cues guide advantageous instrumental behaviors, they also may support the development of detrimental behaviors such as drug abuse. Indeed, cues frequently paired with drug use (e.g., drug paraphernalia) develop incentive value that in turn promotes drug seeking and contributes to the relapse [see review in Ref. (6)]. Given the formidable influence of incentive cues on both positive and negative behaviors, it is important to better understand the neurobiological mechanisms subserving cue-driven behaviors. One system repeatedly implicated in incentive motivational processes is the mesolimbic dopamine (DA) system (7).

DAergic cell bodies resident to the ventral tegmental area (VTA) send their diffuse projections to various cortical and limbic regions such as the prefrontal cortex, amygdala, hippocampus, and the ventral striatum (nucleus accumbens, NAcc) (8). Together, this network comprises the mesolimbic DA system, a system that is highly implicated in the development and maintenance of reward seeking behaviors. A wide body of evidence supports a role for mesolimbic DA in reinforcement learning and motivation for incentive stimuli (9–12). For instance, natural reinforcers such as food and water, as well as drugs of abuse and brain stimulation reward (BSR), support operant behaviors through their ability to activate the mesolimbic system (13–17). Evidencing this view, DAergic lesions or antagonism attenuate approaches toward, or responding for, these reinforcers (18–21).

Single-unit recording data show that VTA DAergic neurons fire in two distinct modes: low-frequency (1–5 Hz) tonic activity and high-frequency (≥20 Hz) phasic activity, the latter of which is characterized by transient rapid bursts (<1 s) of cell firing (22). In the absence of salient stimuli midbrain DA neurons exhibit tonic pacemaker activity producing a steady DAergic “tone” on high-affinity inhibitory D2-like (D2, D3, D4) DA receptors of the mesolimbic system (23–25). This baseline DAergic tone is believed to facilitate long-term depression (LTD) at cortico-striatal synapses and suppress activity of the basal ganglia indirect pathway (26, 27). The presentation of motivationally relevant stimuli such as primary rewards, however, results in rapid burst firing of midbrain DA cells, which increases terminal DA sufficiently to occupy low-affinity excitatory D1-like (D1 and D5) receptors (24). D1 receptor activation following reward-related stimuli is proposed to result in enhanced long-term potentiation (LTP) at excitatory synapses and activation of the basal ganglia direct pathway, thereby motivating behavior (28). Therefore distinct patterns of DAergic cell activity provide a mechanism through which high- and low-affinity DA receptor populations may be differentially activated resulting in the conveyance of distinct reward-related information.

Interestingly, DA neurons adapt to reward presentations by signaling the difference in value of “expected” versus “received” rewards, or a reward prediction error (29, 30). In support of this theory, the presentation of an unexpected reward, such as reinforcer delivery in the initial stages of a conditioning paradigm, results in burst firing of midbrain DA neurons. Following repeated presentations, however, this phasic DA signal previously coupled to reward receipt now results from presentation of reward-predictive stimuli that precede reward delivery (29, 31). The magnitude of burst activity is greater to the reward-predictive cues when the probability of reward is high, but when the probability of reward is low DAergic cell activity is greater during reward receipt. Conversely, midbrain DA neurons cease their firing when no reward or an aversive stimulus is delivered, communicating a negative reward prediction error (30). It should be mentioned that non-appetitive auditory, tactile, or visual sensory stimuli can result in burst firing of midbrain DA cells (32–35), however, these cells appear to fire preferentially to reward-related stimuli (36). Thus, phasic activation of midbrain DA neurons transmits information about previous (expectancy) and current reward situations, making this form of signaling particularly important to the development of conditioned reward associations (37, 38). Indeed, Zweifel et al. (39) reported that genetic inactivation of *N*-methyl-d-aspartate (NMDA) glutamate receptors on DA neurons, a treatment that blocks the ability of DA neurons to burst fire, attenuates stimulus-response learning.

## Measurement of Extracellular Dopamine Concentrations within the NAcc

While electrophysiological recordings provide valuable information about DAergic cell activity patterns, the relationship between DA cell firing and neurotransmitter release at terminal regions is not linear (40). Therefore, techniques that allow for the measurement of extracellular DA concentrations within discrete structures are critical to evaluate functional roles of regional DA release.

Tonic DA levels can be measured using *in vivo* microdialysis techniques, allowing for neurochemical analysis of brain dialysate with a temporal resolution of minutes [(41), for review see Ref. (42)]. A wealth of microdialysis data correlate reward-related phenomena with enhanced DA levels at mesolimbic terminal regions, such as the NAcc. For example, DA levels are elevated in target regions of the mesolimbic system following self-administration of either food (14, 15, 43), water (17), or drugs of abuse (44–50). However, a sample rate of minutes is insufficient to disentangle DA release related to reward receipt versus cue-evoked DA. Direct assessment of subsecond fluctuations in DA concentration due to phasic firing requires the use of techniques with greater temporal resolution, such as fast-scan cyclic voltammetry (FSCV). FSCV has consistently been utilized to measure subsecond transient changes in DA concentration within distinct brain areas [for review see Ref. (51)] of both anesthetized (52) and behaving animals (53, 54). However, FSCV cannot readily differentiate between norepinephrine and DA signals. Thus, voltammetric assessment of phasic DA activity is best suited for regions with low noradrenergic input, i.e., the NAcc.

Research employing FSCV demonstrates that stimuli promoting burst activity of DA neurons also produce transient increases in extracellular DA concentration (termed “transients”) at terminal fields of the mesolimbic system. For example, several studies show enhanced DA transient activity within the NAcc coincident with the presentation of a food reward or related reward-predictive cues (55–59) – stimuli known to result in phasic burst firing of midbrain DA neurons (30, 60, 61). Importantly, a wide body of FSCV data support a role for reward-evoked striatal DA as a reward prediction error signal. Indeed, enhanced phasic DA transmission is reliably observed following unexpected reward delivery or, after conditioning, in response to cues that predict reward (40, 55, 57, 59, 62–64). Further, in congruence with electrophysiological data, reward omission or administration of an aversive stimulus results in decreased extracellular DA within the ventral striatum (65–67).

Shifts in midbrain DA neuron activation from tonic low-frequency activity to phasic high-frequency burst firing likely result from changes in synaptic input from glutamate and gamma-aminobutyric (GABA) afferents to VTA DA cells. The VTA receives excitatory afferents from both sensory and cognitive regions, including glutamatergic afferents from the prefrontal cortex, the extended amygdala, and the laterodorsal and pedunculopontine tegmental nuclei (68–70) and inhibitory GABAergic input from the basal ganglia and rostromedial tegmental nucleus. DAergic neurons in brain slice preparations (i.e., without afferent input) exhibit pacemaker-like tonic activation but do not fire in bursts, thus DA cells are “conditional” rather than “intrinsic” bursters (71, 72). Indeed, burst firing of DA neurons requires glutamatergic input and the activation of DAergic cell NMDA glutamate receptors (73, 74). Conversely, GABAergic input to midbrain DA neurons dampens burst firing and returns the cell to baseline pacemaker-like activity (75). Thus, the maintenance of midbrain DAergic firing patterns requires a balance between excitatory and inhibitory VTA afferent signals. A key signaling network implicated in the maintenance of this balance is the endocannabinoid system.

## A Brief Overview of the Endocannabinoid System

The endocannabinoid system, composed of endogenous cannabinoids (i.e., endocannabinoids), cannabinoid receptors, and the enzymes responsible for endocannabinoid synthesis and degradation, is a neuromodulatory network known to play a role in a number of neural processes, including learning and memory, motivation, reward, operant behavior, and neuroplasticity (76–79). Cannabinoid receptors were the first components of this system to be discovered. In the early 1990s, Δ^9^-tetrahydrocannabinol (THC), the primary psychoactive constituent of the cannabis plant, was found to produce its characteristic effects as a partial agonist of a G-protein-coupled receptor (GPCR) (G_i/o_) isolated from neural cell lines and later named cannabinoid receptor type 1 (CB1) (80, 81). A few years after this discovery, a second cannabinoid receptor (cannabinoid receptor type 2; CB2) was identified (82). CB1 and CB2 receptors differ in anatomical distribution as well as function. Autoradiography studies show that CB1 is the primary cannabinoid receptor of the central nervous system (CNS) with a wide distribution throughout the brain and periphery and the highest concentrations of CB1 binding found in brain regions implicated in the actions of cannabis (83–85). Conversely, CB2 is more abundant in the periphery and expressed primarily in immune cells, including microglia of the CNS (86), and is thus believed to primarily play a role in immune function (87). Recent evidence, however, also supports a role for CB2 in a variety of neurological processes, such as anxiety, pain, and addiction (88–91). It is important to note that while this review will focus on activity at CB receptors, endocannabinoids also interact with various ligand-gated ion channels (e.g., vanilloid receptor type 1 channels) as well as other GPCRs, such as GPR55 (92, 93).

**Figure 1:**
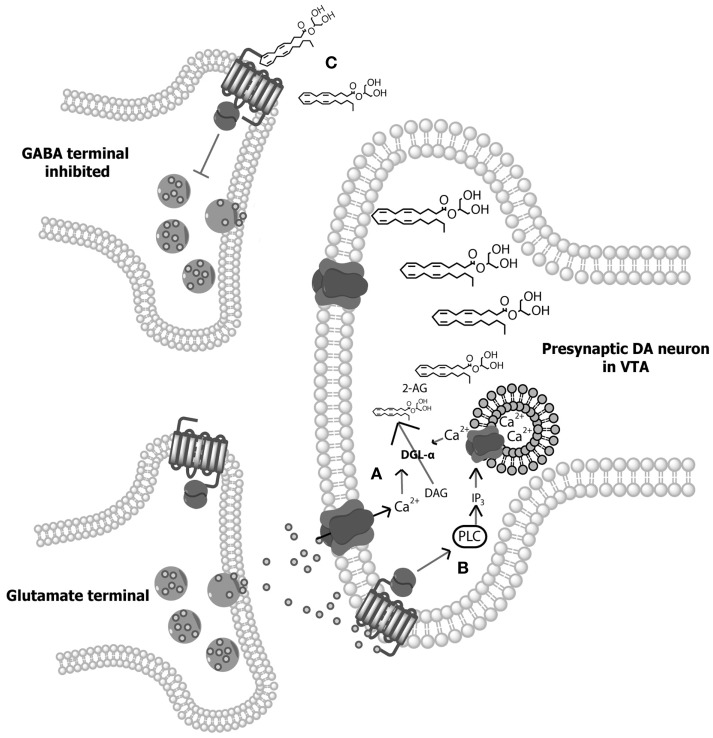
**Illustration of 2-arachidonylglycerol (2-AG) synthesis**. **(A)** Depolarization-induced Ca^2+^ influx within dopamine (DA) neurons of the ventral tegmental area (VTA) results in the hydrolysis of 1,2-diacylglycerol (DAG) by DGL-α and DGL-β lipases to form 2-AG (98, 100). **(B)** Alternatively, activation of G_q/11_ protein-coupled receptors (e.g., group 1 metabotropic glutamate receptors) directly stimulate phospholipase-Cβ (PLC), resulting in the hydrolysis of membrane phosphate phosphatidylinositol 4,5-bisphosphate (PIP2) to DAG, allowing for subsequent hydrolysis of DAG to 2-AG (101–103). In addition, Ca^2+^-dependent and GPCR-dependent 2-AG synthesis can co-occur to synergistically produce high concentrations of 2-AG (104, 105). **(C)** Following on-demand synthesis, 2-AG then diffuses from the postsynaptic DA neurons and binds with CB1 receptors on presynaptic gamma- aminobutyric acid (GABA) cells, inhibiting GABA release and thereby disinhibiting DAergic cell activity.

The discovery of cannabinoid receptors was followed shortly after by the identification of their primary endogenous ligands – *N*-arachidonylethanolamine (anandamide; AEA), a partial agonist at CB1 receptors, and 2-arachidonylglycerol (2-AG), a full agonist at both CB1 and CB2 receptors (94–96). The biosynthesis of AEA is not fully understood, although it is generally agreed that AEA is synthesized from *N*-arachidonoyl phosphatidylethanolamine (NAPE) in a Ca^2+^-dependent manner via one of the several possible enzymatic pathways (97, 98). Conversely, both Ca^2+^-dependent and Ca^2+^-independent synthesis pathways for 2-AG have been outlined (illustrated in Figure 1). Following synthesis and release, AEA and 2-AG signaling is quickly terminated through cellular reuptake and hydrolysis primarily by the enzymes fatty acid amide hydrolase (FAAH) and monoacylglycerol lipase (MAGL), respectively [FAAH can also hydrolyze 2-AG (99)]. While several additional endocannabinoids have since been discovered, AEA and 2-AG remain the best characterized.

The most prominent physiological role for endocannabinoids is as synaptic retrograde messengers – molecules that transmit messages from post- to presynaptic cells. Indeed, both AEA and 2-AG signal retrogradely. Like all known endocannabinoids, AEA and 2-AG are lipid molecules, which in contrast to classic neurotransmitters, are synthesized and released from neurons “on demand” upon stimulation (106, 107). These lipophilic messengers diffuse through the postsynaptic membrane and interact with cannabinoid receptors of nearby cells. Both CB1 and CB2 receptors are G_i/o_-coupled receptors; therefore, their activation results in a decrease in cAMP activity within the cell and subsequent inhibition of neurotransmitter release, resulting in negative feedback to presynaptic cells (108–110). When Ca^2+^-mediated, this process has been termed depolarization-induced suppression of inhibition (DSI) or excitation (DSE) depending on the neurotransmitter system being inhibited (111), and works to modulate synaptic plasticity. Research on the individual functions of AEA and 2-AG suggests that 2-AG is the principal endocannabinoid involved in DSI- and DSE-induced plasticity (109).

## Endocannabinoid Modulation of Dopamine Transmission

Like the discovery of the endocannabinoid system, the ability of endocannabinoids to modulate mesolimbic DA transmission was uncovered through investigations into the effects of THC. All drugs of abuse increase DA neurotransmission in the NAcc (112, 113) and although some early studies exploring a role for DA in cannabinoid abuse potential yielded incongruent results [e.g., Ref. (114, 115)], today a large body of evidence suggests that marijuana is no exception. Indeed, cannabinoid administration enhances extracellular DA concentrations in the ventral striatum (116–119). Further, this effect is dependent on CB1 receptor signaling, as pretreatment with the CB1 receptor antagonist/inverse agonist SR141716A (rimonabant) blocks the ability of THC, or the synthetic cannabinoid receptor agonist WIN 55,212-2 (WIN), to enhance striatal DA levels (119). Seminal single-unit recording data from French et al. (120) show that cannabinoids enhance extracellular DA concentrations in the NAcc through increasing both the baseline firing rates and burst frequency of midbrain DA neurons. These enhancements in VTA DA cell firing are also CB1-dependent as they are attenuated by rimonabant (121).

Interestingly, while midbrain DA neurons release endocannabinoids during phasic activation, they do not express CB1 receptors (83, 109, 122, 123). This suggests that cannabinoids excite VTA DA cells via an indirect mechanism. Aside from DAergic cell bodies, the VTA also contains GABAergic neurons that inhibit midbrain DA activity through interaction with GABA_A_ receptors on DA cells (8). The application of the GABA_A_ agonist bicuculine to VTA-containing slices enhances DAergic cell activity (124). Thus, cannabinoids may increase DA neuron burst firing through the inhibition of VTA GABA cells, resulting in disinhibition of midbrain DA neurons (109, 110). In support of this explanation, Szabo et al. (125) found that WIN reduces electrically evoked GABA_A_-mediated inhibitory postsynaptic currents in DA neurons of VTA slices, and these effects are blocked by rimonabant. These data led Lupica and Riegel (110, 123) to propose a model of endocannabinoid-dopamine system interaction wherein enhanced activation of VTA DA neurons promotes release of endocannabinoids, which then activate presynaptic CB1 receptors on GABA terminals, resulting in DSI (illustrated in Figure 1). In line with this model, rimonabant administration attenuates the typical enhancement of DA transient concentrations in the NAcc seen following cocaine administration (64).

## The Role of Endocannabinoids in Cue-Motivated Behaviors

As discussed above, burst firing of DAergic neurons plays an integral role in stimulus-response learning (39, 55, 58, 59). Therefore, given the well-documented ability of endocannabinoids to modulate phasic DA signaling, it stands to reason that endocannabinoids are also involved in modulating incentive motivation. Much research utilizing cue-induced reinstatement of operant behaviors illustrates the involvement of the endocannabinoid system in various aspects of cue-induced responding. In this model, an established operant behavior is extinguished and then reinstated following the presentation of stimuli previously paired with reinforcer availability. Thus incentive cues work to reinvigorate reward-seeking in the absence of reward. Utilization of this model shows that while systemic administration of rimonabant does not affect operant responding for palatable stimuli (126), it attenuates cue-induced reinstatement of food-seeking behavior (127–129). Given that rimonabant also blocks the development of conditioned place preference for food (130), these data suggest that endocannabinoid signaling at CB1 receptors modulates the incentive value of food-associated stimuli. Indeed, the CB1 receptor antagonist AM251 attenuates cue-maintained responding for food under a second-order schedule of reinforcement (131). These effects do not seem to be reinforcer specific as endocannabinoid signaling is critical for cue-induced reinstatement of drug-seeking behavior. Systemic rimonabant attenuates cue-induced reinstatement of cocaine (126), heroin (132), methamphetamine (133), alcohol (134), nicotine (127), and THC (135). Further, the effects of CB1 antagonism on cue-induced reinstatement of reward seeking may be dependent on DAergic mechanisms, as DA antagonism also blocks cue-induced reinstatement of reward seeking behavior (136, 137).

In recent research from our laboratory, Oleson et al. (138) employed an intracranial self-stimulation (ICSS) task along with FSCV to investigate endocannabinoid-mediated disinhibition of cue-evoked DA. In this procedure, a light and tone cue signaled reinforcer availability and each operant lever press resulted in the delivery of electrical BSR to the VTA. This work replicated previous findings showing that over the course of operant training as task performance improves, NAcc DA transients time lock to cues that predicted reward (in this case BSR) availability (139). However, either intravenous (i.v.) or intra-VTA delivery of rimonabant dose-dependently increased response latency while simultaneously disrupting cue-evoked DA transients within the ventral striatum, supporting a role for specifically midbrain DAergic activity in the encoding of reward-related cues. Further, similar results were found in rats responding for food reward, suggesting that the involvement of endocannabinoids in cued responding is not reinforcer specific. Next, URB597, a FAAH inhibitor, and JZL184, an inhibitor of MAGL, were utilized to increase levels of either AEA or 2-AG, respectively. While pretreatment with URB597 (i.v.) had no effect on cued ICSS responding, both i.v. and intra-VTA administration of JZL184 decreased response latency and enhanced cue-evoked accumbal DA transmission. Further, the effects of JZL184 on ICSS responding and DAergic transmission were blocked by pretreatment with a sub-threshold dose of rimonabant, suggesting efficacy through a CB1-dependent mechanism. Taken together, these data indicate that endocannabinoids, specifically 2-AG, modulate DAergic encoding of environmental cues to control reinforcement-directed behaviors.

## Endocannabinoids and Time as a Discriminative Cue

While the perception and processing of external environmental stimuli directs reward-seeking behaviors, organisms also rely on interoceptive cues (e.g., hunger or thirst) in deciding how to interact with their environment. One such internal signal, which has recently received a great deal of attention, is time estimation. Indeed, organisms rely on internal “biological clocks” to coordinate behaviors from the microsecond processing of fluid movements to the daily rhythms of the sleep–wake cycle. Of particular relevance to reward-motivated behaviors is the timing of intervals in the seconds-to-minutes range involved in a number of fundamental behaviors such as reward seeking and decision making (140). Like the processing of external reward-predictive stimuli, internal processing of temporal information is mediated by DAergic systems. Disorders that result in disruption of DA function, such as Parkinson’s disease (141, 142) or Huntington’s disease (140), or the administration of DA antagonists (e.g., haloperidol) slows the internal clock resulting in the perception that time is passing much faster (in comparison to internal temporal representations) and consequent overestimation of interval time (143–145). Conversely, disorders associated with augmented DA levels [e.g., schizophrenia (146)], or the administration of drugs that agonize DAergic signaling (e.g., cocaine and amphetamine) speeds up the internal clock leading to the perception that time is passing more slowly and resulting in underestimation of interval time (147–149). Similarly, cannabis users report a slowing of the subjective experience of time (150, 151), presumably related to the ability of cannabinoids to enhance DA transmission. These findings have been replicated in animals with both THC and WIN administration decreasing time sensitivity in both temporal discrimination (152) and estimation (153) tasks. Importantly, Han and Robinson (153) found that administration of rimonabant disrupts interval estimation and delays operant responding, suggesting a role for endocannabinoids in interval timing.

Oleson et al. (154) utilized a fixed-interval (FI) schedule of food reinforcement to uncover the role of endocannabinoid modulation of phasic DA signaling in interval timing. In FI schedules, operant behaviors are reinforced on the basis of time (155). These schedules are known to produce a behavioral pattern known as an FI “scallop,” wherein rates of responding accelerate over the course of the interval until reaching a peak response rate just before reward delivery (155). In this investigation, Oleson et al. (154) showed that phasic DA release in the mouse NAcc is inversely related to interval time; i.e., the start of the interval is characterized by a high frequency of phasic DA release events (resulting in enhanced DA concentration) which then gradually decrease in frequency as the interval continues, finally reaching a DA concentration minimum at interval terminus. These data thus support a role for DA in encoding of the principal cue predicting reinforcer availability during FI schedules – time. The endocannabinoid system was subsequently shown to modulate these effects as systemic administration of WIN dose-dependently increased the pattern of DA release and accelerated the temporal pattern of responding. These results were mimicked by intraperitoneal (i.p.) administration of JZL184, but not by i.p. URB597. Further, the effects of JZL184 were CB1-dependent as pretreatment with AM251 blocked 2-AG-induced increases in DA concentration as well as elevations in response rate and thus normalized behavior to the typical FI scallop response pattern. Although both DAergic and endocannabinoid systems have been independently implicated in the temporal control of behavior (153, 156, 157), these data represent the first report showing how phasic striatal DA may work to signal interval duration and the crucial role for endocannabinoid signaling in the encoding of this interoceptive time cue.

Oleson et al. (154) posit that the high DA concentrations seen at the start of the interval and the low DA concentrations observed at interval terminus, promote reinforcement-driven motivation resulting in the initiation of regulated operant responding and increased lever pressing, respectively. Conversely, moderate levels of DA, characteristic of the middle of the interval, promote engagement in adjunctive behaviors – behaviors that do not result in reinforcement delivery, measured as inactive lever presses. Their data align well with current theories on basal ganglia function (158). These theories suggest that high concentration DA surges arriving in the NAcc activate D1 receptors on medium spiny neurons (MSNs) comprising the direct pathway and promote action sequences (e.g., lever pressing). Baseline moderate concentration DA signals, however, are believed to activate the indirect pathway through interaction with D2 receptors on MSNs and work to inhibit primary reward-directed action sequences in favor of alternative, perhaps exploratory, adjunctive behaviors (24, 159). Thus, fluctuations in NAcc DA concentration direct reward-related behavior, likely through the modulation of striatal afferent inputs (160, 161). Enhancement of CB1 activation through administration of WIN or JZL184 increased both NAcc DA concentration and response rate, suggesting that activation of the endocannabinoid system drives direct pathway activation and primary reward seeking. Interestingly, both enhancement of 2-AG transmission (with JZL184) and disruption of CB1 activation (with AM251) attenuated adjunctive behaviors, supporting a role for the endocannabinoid system, not only in the direction of primary-reward driven behaviors governed by environmental cues but also the directions of adjunctive behaviors when environmental cues dictate that reward is not available. However, in opposition to this view, a recent report by Cui et al. (162) suggests a departure from the classical interpretation of basal ganglia function and supports a role for MSNs of both the direct and indirect pathway in action initiation. In this investigation, Cui et al. (162) utilized Cre-dependent viral expression of genetically encoded calcium indicators in transgenic mice along with time-correlated single photon counting to individually quantify D1-expressing and D2-expressing striatal MSN activation during an operant lever pressing task for food. They found that MSNs of both pathways were transiently activated just prior to the initiation of contralateral movements and quiescent during periods when the animal was not moving, suggesting that activation of both the direct and the indirect pathway promote behavioral output. Therefore, it may be that over a fixed time interval, fluctuation in extracellular DA preferentially excites individual cells within both pathways, which work together to coordinate either primary reward seeking or adjunctive behaviors during FI schedules.

## The Striatal Beat Frequency Model and Endocannabinoid Modulation of Timing

The question remains, however, how does the DAergic system signal the occurrence of a distinct temporal window in order to represent an interoceptive time cue? One convincing model on the neurobiology of interval timing is the striatal beat frequency (SBF) model (148, 163). SBF postulates that cortico-striatal circuits encode interval durations of seconds to minutes, and these circuits are coordinated by midbrain DAergic input. Within the frontal cortex, neurons oscillating in the alpha range (8–13 Hz) act as an internal clock (164). At interval onset, the presentation of a distinct conditioned stimulus results in phasic firing of midbrain DA neurons and these transient surges of extracellular DA synchronize oscillating neurons in the frontal cortex, likely through inhibition of desynchronized cells (163). However, briefly after synchronization, cortical neurons begin to fall out of phase with one another, returning to their inherent individual periodicities. Thus, distinct interval durations are marked by unique patterns of neuronal ensembles firing in-phase. These cortical oscillators synapse onto MSNs of the striatum that function as “coincidence detectors” – connecting distinct patterns of oscillator activity with the occurrence of external stimuli (e.g., reward delivery). Striatal MSNs are uniquely situated to detect coincident neuronal activity with each MSN receiving input from 10,000 to 30,000 different thalamic and cortical neurons (165). Further, striatal MSNs exhibit both highly polarized “down” states (−90 mV) and less polarized “up” states (approximately −60 mV) (166) with transitions from down to up states requiring either VTA DA input (26, 167) or excitatory glutamatergic input from other cortical or subcortical structures (166, 168). High levels of DAergic input to the striatum at interval onset are hypothesized to “clear out” irrelevant information within the coincidence detector by hyperpolarizing striatal cells into their down state (148). Conversely, phasic surges of extracellular DA within the striatum at reward delivery bring striatal cells into their up state and promote LTP at active cortico-striatal synapses (27, 163, 169). Therefore, the pattern of cortical inputs spiking at the time of reinforcement, which represents a unique population code for the time elapsed since interval initiation, will undergo Hebbian strengthening. LTP at these striatal synapses allows the organism to learn specific interval durations and initiate reward seeking when a matching pattern of cortical efferent activity is encountered.

While the SBF model represents a neurobiologically plausible framework within which to examine interval timing, there are inconsistencies in this model. For example: how does phasic DA at cue onset hyperpolarize striatal cells given that D1 stimulation is excitatory (i.e., G_s_-coupled)? One possibility is through collateral inhibition wherein D1-mediated excitation in specific MSNs leads to a net inhibition of striatal cells through a lateral inhibitory feedback network (170–172); however, this has yet to be explored within interval timing tasks. Further, there has been no direct voltammetric assessment of striatal DA levels and consequent induction of striatal LTP mechanisms.

The voltammetric data presented by Oleson et al. (154) lend support to the SBF model. In their investigation, extracellular DA is high at the start of the interval, providing a mechanism through which NAcc cells may be hyperpolarized (perhaps through collateral-driven lateral inhibition) and the coincidence detector reset. As the interval progresses DA levels gradually decrease, likely releasing striatal cells from collateral feedback inhibition and allowing for DA transient-induced cortico-striatal LTP. It remains unclear, however, how DA levels gradually decrease as the interval continues. This may occur through striatal cell ramping activity. Indeed, a subset of NAcc neurons display a ramping activity pattern during interval estimation wherein firing rates linearly increase from CS presentation and peak at the time of reward expectation (173, 174). The accumbens sends direct inhibitory input to the VTA (175), thus ramping activity may result in progressive DA decline from CS presentation (176). Data from Oleson et al. (154), however, do not determine if NAcc DA activity is integral for interval estimation, but, rather, provide evidence for a correlation between accumbal phasic DA activity and interval estimation. Future investigations are required to determine if phasic NAcc DA release is required for interval estimation.

The SBF model suggests a specific role for the dorsal striatum in temporal processing, citing the ability of dorsal, but not ventral, striatal lesions to impair temporal control (177, 178). However, lesions of the NAcc core disrupt timing of Pavlovian responses, as evidenced by decreased approach to a food receptacle at CS+ presentation (179). Further, while Galtress and Kirkpatrick (180) found that accumbal lesions do not disrupt the tracking of temporal windows in a peak-interval (PI) timing procedure, animals do exhibit a decreased ability to modify behavior when an expected reward is not delivered at interval terminus. PI is an extension of an FI schedule with the addition of non-reinforced probe trials which result in Gaussian response curves characterized by increased operant responding up to the point of expected reward followed by a gradual decrease in responding. Thus, the NAcc core likely functions to integrate temporal cues specifically with reward value and availability. Given that responding during an FI combines both temporal estimation and expectations regarding reinforcer value, the role of the NAcc core in operant responding during FI schedules of reinforcement merits more examination. Finally, it should be noted that while recordings of phasic NAcc DA activity support the SBF model, no voltammetric recordings during FI responding have been taken in the frontal cortex due to the high levels of cortical noradrenergic innervation. However, recent optogenetic investigations show that disruption of D1 receptor transmission within the prefrontal cortex impairs performance on an FI task (157), supporting an integral role for phasic DA within the frontal cortex in interval timing.

**Figure 2:**
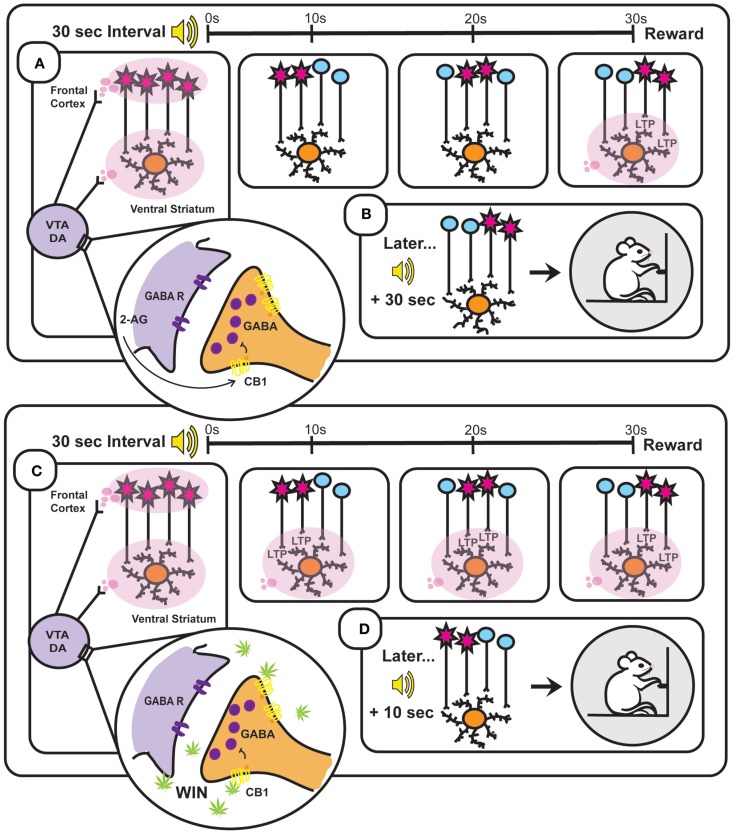
**Based on the striatal beat frequency model of interval timing – a schematic representation of the neurobiology underlying interval timing during a typical 30 s fixed interval (A,B) and during a 30 s fixed interval following administration of the synthetic cannabinoid WIN 55,212-2 (WIN) (C,D)**. **(A)** At interval onset, phasic dopamine (DA) transmission resets the internal clock through synchronization of frontal cortical oscillators [depicted in **(A)** as simultaneously firing cells and illustrated as red cell bodies] and clears out the coincidence detector (ventral striatum). These phasic signals arise through burst firing of ventral tegmental area (VTA) DA cells, which is facilitated by endocannabinoid (2-AG)-mediated suppression of GABA release onto VTA DA neurons. As the 30 s interval progresses (illustrated in the three panels to the right depicting time points at 10, 20, and 30 s), the once synchronized cortical oscillators fall out of phase with one another at a reliable rate. Reward delivery at interval terminus results in phasic DA transmission within the ventral striatum that enhances LTP at active cortico-striatal synapses. **(B)** Later when the same (in this case auditory) stimulus signals interval onset, cortical oscillators exhibit characteristic periodicities and when the previously strengthened synaptic pattern active at interval terminus is encountered again (30 s after interval onset) its activation will promote reward seeking. **(C)** WIN administration results in phasic activation of VTA DA neurons through binding to CB1 receptors on VTA GABAergic neurons and thereby disinhibiting VTA DA transmission. Drug-induced aberrant DAergic activation throughout the interval induces LTP at cortico-striatal synapses active prior to reward delivery (illustrated in the three panels to the right depicting time points at 10, 20, and 30 s). **(D)** This Hebbian strengthening of synaptic activity characteristic of earlier time points within the interval promotes premature reward seeking following subsequent cue presentation.

The ability of cannabinoids, as well as other drugs that augment DAergic signaling, to enhance internal clock speed has been well documented (181–184), however, the neurobiological mechanisms underlying this effect remains unclear. Within the framework of the SBF model, enhancement of phasic DA following cannabinoid administration may affect time perception through agonizing DA transmission within the striatum. Indeed, selective manipulation of DA levels within the ventral striatum disrupts performance in timing-dependent operant tasks (185–187). Further, direct administration of DA into the NAcc enhances internal clock speed (188). Increased phasic DAergic activity in the NAcc core, as seen following WIN administration (154), may result in induction of LTP at cortico-striatal synapses that are active much earlier in the interval than those active at reinforcer delivery. This abnormal “stamping in” of premature interval estimations would thus promote reward seeking prior to interval terminus, likely through activation of the basal ganglia’s direct pathway [but see Ref. (162)] (Figure 2). However, future investigations are necessary to determine if cortico-striatal LTP adheres to specific patterns during interval estimation tasks, and examine how these patterns are changed following DAergic agonist administration.

A greater understanding of the role of phasic DA in interval timing is integral to the study of drug addiction. Individuals that are more sensitive to the time-altering effects of drug administration are also more sensitive to the experience of stimulant-induced euphoria, suggesting that similar brain pathways maintain internal clock speed and drug reward (189). Indeed, drugs of abuse increase extracellular DA within the ventral striatum and have the ability to hasten internal clock speed. Therefore, augmentation of internal clock representation may underlie a path through which drugs alter stimulus-reward associations and promote inappropriate reward seeking, such as that seen in delay discounting paradigms. Delay discounting is a maladaptive decision-making strategy characteristic to addiction wherein individuals show a preference for smaller/immediate over larger/delayed rewards. This temporal shift in reward seeking is generally considered an indication of impulsivity, but one could also imagine delay discounting to result from augmentation of internal clock representations. Certainly, a number of human studies indicate a positive correlation between substance abuse, including marijuana abuse, and measures of delay discounting (190–196). Investigations into the neurobiology of this phenomenon implicate alterations in striatal DA activity (197, 198), suggesting a link between drug-induced enhancement of phasic DA signaling and temporally biased reward seeking. Ostlund et al. (199) showed that repeated cocaine exposure enhances phasic DA transmission and increases cue-evoked food seeking. Thus, enhanced accumbal DA in response to reward-predictive cues following drug use, may, through interaction with the proposed SBF timing neurocircuitry, accelerate the internal clock and promote the choice of immediate over delayed rewards. Importantly, Hernandez et al. (198) showed that systemic pretreatment with rimonabant attenuates cocaine-induced delay discounting. These findings support a role for endocannabinoid-modulated enhancement of phasic DA transmission following drug use and subsequent temporal shifts in reward seeking resulting from an enhancement of internal clock speed. However, it remains unclear if decision making during delay discounting paradigms occurs within the same timescale as that of interval estimation, or relies on strategies adopted prior to task onset.

## Conclusion

A wide body of evidence supports a role for endocannabinoid modulation of phasic midbrain DAergic activity. Phasic burst activation of DA neurons can be measured as transient fluctuations of extracellular DA in terminal regions of the mesolimbic system (e.g., the NAcc) using FSCV. In congruence with reward prediction error, following repeated stimulus-reward pairings, reward-predictive cues result in burst firing of mesolimbic DA neurons and transient increases in NAcc DA levels. This transient burst of striatal DA likely promotes maintenance of reward-seeking behaviors through activation of the basal ganglia pathways. In addition to tracking of external reward-related cues, phasic changes in striatal DA encode interoceptive cues, such as interval time that allow for the coordination of goal-directed responding. In support of this view, drugs that enhance striatal DA levels also increase individuals’ internal clock thereby resulting in the perception that time is passing more slowly. Further, animal studies show that administration of the CB1/CB2 agonist WIN or the 2-AG degradation inhibitor JZL184 enhances NAcc core DA levels and promotes premature reward seeking in a FI task. The precise neurobiological mechanism(s) through which these effects occur, however, remains unknown. The SBF model of interval timing provides a unique framework with which to examine the role of phasic DA in interval timing. SBF posits that striatal DA fluctuations mediate interval timing through selectively promoting LTP at cortico-striatal synapses active at specific interval durations. Thus, DAergic disruption of interval timing may occur through aberrant induction of LTP at cortico-striatal synapses representative of premature interval lengths. Given the ability of drugs of abuse to potently augment NAcc DA levels, it is not surprising that drug administration also disrupts interval estimation. Alterations in interval timing may also underlie addiction-related behaviors such as delay discounting, which is enhanced by endocannabinoid-mediated increases in striatal DA. A conserved mechanism in interval timing and impulsive choice suggests that following drug use, enhanced delayed discounting is consequential to augmentation of one’s internal clock, which results in temporal-biasing of reward-seeking at the presentation of reward-related cues.

## Conflict of Interest Statement

The authors declare that the research was conducted in the absence of any commercial or financial relationships that could be construed as a potential conflict of interest.
